# Outcomes after surgical treatment of acetabular fractures: a review

**DOI:** 10.1186/s13037-019-0196-2

**Published:** 2019-03-16

**Authors:** Navid Ziran, Gillian L. S. Soles, Joel M. Matta

**Affiliations:** 1 St. Joseph’s Hospital and Medical Center, Creighton University School of Medicine, 500 W. Thomas Road, Suite 850, Phoenix, Arizona 85013 USA; 2 Department of Orthopaedics, Strong Memorial Hospital, University of Rochester, 601 Elmwood Avenue, Rochester, New York 14620 USA; 3 The Steadman Clinic, 181 West Meadow Drive Suite 400, Vail, Colorado 81657 USA

**Keywords:** Acetabular fracture, Hip joint, Post-traumatic arthritis, Surgical fixation, Survivorship

## Abstract

Acetabular fractures are fractures that extend into the hip joint and pose a challenge for orthopaedic trauma surgeons. The first known descriptions of surgical fixation of acetabular fractures were case reports in 1943. In 1964, Robert Judet, Jean Judet, and Émile Letournel published a landmark article describing a classification system and surgical approaches to treat acetabular fractures. These teachings had a significant effect on clinical outcomes after surgical fixation of acetabular fractures. In 1980, Letournel demonstrated 80% good-to-excellent results in 492 hips, and in 2012, Joel Matta demonstrated 79% survivorship in 816 patients follow surgical acetabular fixation. Both Letournel and Matta have definitively shown that anatomic reduction of the fracture is the most influential factor predictive of clinical outcome. The intent of this review is to summarize the salient factors affecting clinical outcomes after surgical treatment of acetabular fractures.

## Introduction

Acetabular fractures are among the most complex injuries treated by orthopaedic surgeons. The work of Robert Judet and Émile Letournel began our understanding of surgical approaches, reduction techniques, complications, and results [1, 2]. Good to excellent functional results have been reported in up to 80% of operatively treated acetabular fractures at 20 years [3]. Multiple factors influence clinical outcome following an acetabular fracture, including pre-existing conditions, injury-related factors, surgical considerations, and postoperative complications. The quality of the articular reduction has been shown to be of utmost importance in determining clinical outcome [3, 4]. This chapter will focus on the factors affecting outcome and the long-term results of operatively treated acetabular fractures.

## Background

The first outcomes published were case reports of acetabular fracture surgical fixation. In 1943, Levine fixed an acetabular fracture via the inner aspect of the ilium with plates and screws [5]. Urist described surgical fixation of an acetabular fracture via an anterior iliofemoral approach in 1949 [6]. The first larger case series was published by Okelberry in 1955 in which he performed internal fixation of seven acetabular fractures via an anterior iliofemoral approach [7]. In 1958, Knight and Smith [8] discussed the increasing frequency of acetabular fractures due to automobile accidents. In an effort to improve outcomes, they described surgical treatment of eight “central” acetabular fractures at the Campbell Clinic using Knowles pins. In this article, they mention “*the primary objective, however, is reduction and fixation of the fractures which involve the weight-bearing vault (from 10 to 3 on the clock).*” They determined that the results after open reduction were superior to “older” methods of manipulation and traction and felt long-term studies of a larger series of patients were warranted.

Rowe and Lowell performed the first large study in 1961 [9]. In this study, they described their outcomes after both operative and non-operative treatment of 93 acetabular fractures with an average follow-up of six-years (range 1–27 years). The authors classified the fractures as non-displaced, posterior, inner-wall, and superior or bursting fracture. Outcome correlated with 1) involvement of the weight-bearing dome (roof), 2) femoral head condition, 3) adequacy of reduction of the dislocation (joint congruency), and 4) joint stability after one year. Dislocations of the femoral head were also described in this paper as central, posterior, and anterior. The term “weight-bearing dome” was used in this paper, although no specific anatomic or radiographic landmarks were defined. Although these terms were informative and provided a starting point for understanding acetabular fractures, they lacked the detail necessary to guide the surgeon’s treatment.

The Rowe and Lowell, article, however, while not discerning the specific fracture patterns, did demonstrate several important points that are still true today. First, they clearly demonstrated that fractures that involve superior areas of the acetabulum have a worse prognosis than involvement of the inferior acetabular area. The “burst” fracture, which was due to “upward and medial thrust” of the femoral head, demonstrated the worst outcomes. Second, they demonstrated that the clinical and radiographic findings of the hip at one year were frequently representative of the long-term prognosis. This early prognostic finding would later be demonstrated by other authors at 1–2 years and will be discussed later in this chapter.

It was not until 1964, when Robert Judet, Jean Judet, and Émile Letournel published the landmark article “Fractures of the Acetabulum, Classification and Surgical Approaches for Open Reduction,” that surgeons had a system to understand and treat these injuries [1]. This article described a methodology to understand, classify, and treat acetabular fractures. This methodology would later prove to have a significant impact on improving surgical outcomes of acetabular fractures. Émile Letournel, Robert Judet’s protégé, expanded on these principles and together, with Judet, published the first edition of Fractures of the Acetabulum in 1981. This book, and its second edition successor published in 1993 [4], became the most widely used reference to understand and treat acetabular fractures. In this text, they also published their outcome findings on 492 operatively-treated acetabular fractures from 1953 to 1989.

Matta later disseminated this knowledge to North America and the rest of the world - where it became an established methodology to understand and treat acetabular fractures. In 2012, Matta published the largest single-surgeon outcome study of operatively-treated acetabular fractures to date [3].

Matta not only disseminated the teachings of Judet and Letournel but expanded upon these reduction and fixation principles. He determined the most important load-bearing portion of the radiographic acetabulum [10, 11]. The primary weight-bearing vector in the acetabulum at mid-stance is 16 degrees medial from the vertical – with an excursion of this primary force vector anteriorly and posteriorly during toe-off and heel strike, respectively [12, 13]. The force on the acetabulum is distributed around this primary force vector [14].

**Fig. 1:**
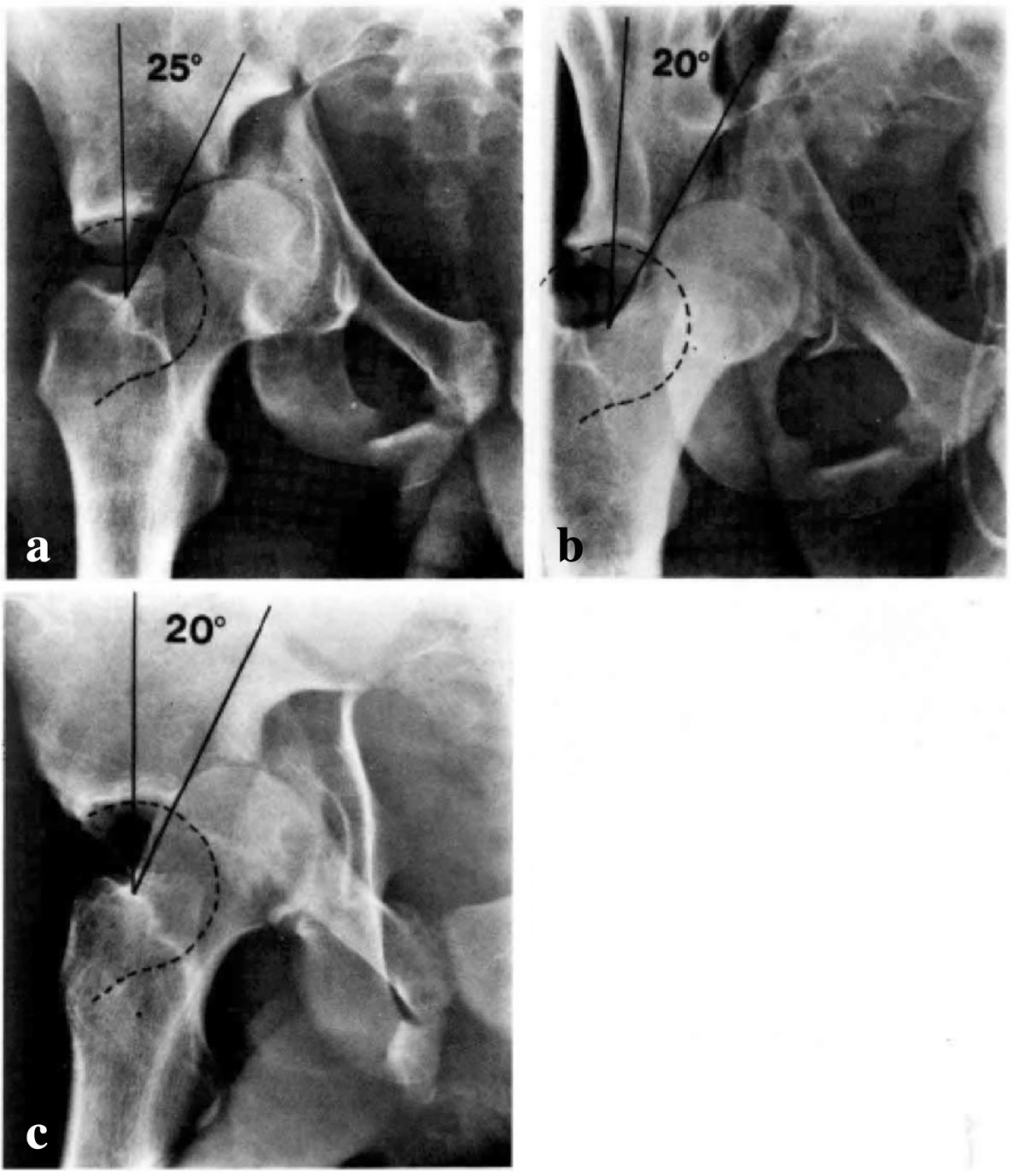
Anteroposterior (**a**), obturator oblique (**b**), and iliac oblique (**c**) view of a T-shaped fracture showing displacement of the femoral head and inadequate roof arc measurement. Surgery is indicated. Permission was granted for utilization of this figure in this review

**Fig. 2:**
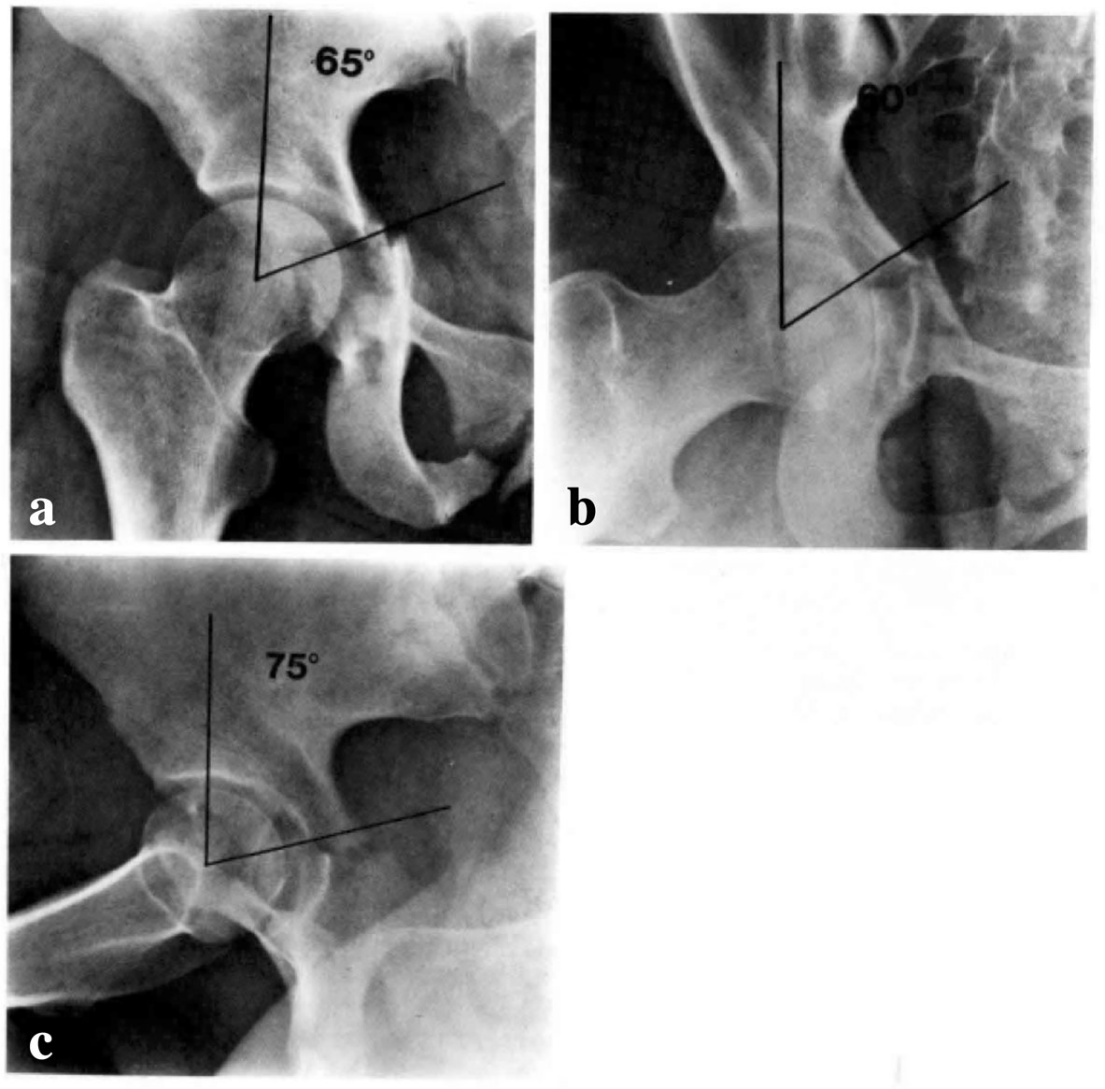
Anteroposterior (**a**), obturator oblique (**b**), and iliac oblique (**c**) view of a T-shaped fracture showing congruence of the femoral head out of traction and adequate roof-arc measurements. Closed treatment is indicated. Permission was granted for utilization of this figure in this review

These areas of force distribution around this vector were designated according to specific “arcs” of the acetabulum based on the different views of the joint. Roof-arc angles are demonstrated in Figs. 1 and 2 and aid in quantitating femoral head congruency with the fractured acetabulum. These measurements were established as a guide for open versus closed management. Note the following minimum roof-arc angles for non-surgical management of acetabular fractures:

- Anterior roof arc > 30
- Medial roof arc > 40
- Posterior roof-arc > 50

These roof-arc measurements are of limited utility in evaluating posterior wall or both-column fractures. In posterior wall fractures, the fracture line is typically displaced posterior to the arc of the acetabulum represented by the posterior roof arc. In both-column fractures, the roof of the acetabulum is separated from the iliac wing and so measurement of this displaced, *rotated*, free fragment has limited value. Matta concluded that:

1. The head of the femur should be congruous with the roof of the acetabulum with the patient out of traction.
2. The anterior, medial, and posterior roof arc angles should be greater than 45 degrees.
3. Roof-arc measurements have limited utility in posterior wall or both-column fractures.

**Fig. 3:**
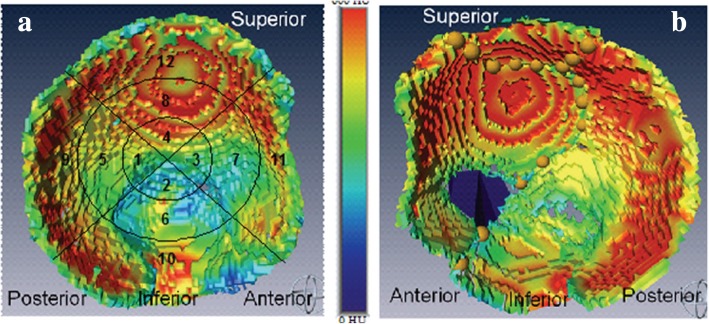
**a** Bone-density mapping of 5 mm of subchdral bone in an intact right acetabulum couresy of Lubovsky et al. 2013. The highest bone density is in the superior and posterior portions of the acetabulum. Injury to these regions tends to correlate with worse prognosis. Density map of a fractured left acetabulum is shown in panel **b**. Fracture lines are marked as yellow circles and a bony defect is shown in dark blue. Permission was granted for figure utilization in this review

More recent studies have attempted to more accurately identify specific zones in the injured acetabulum associated with poorer prognosis. In one particular study, twenty-four patients with acetabular fractures were analyzed [15]. Pre-operative computed tomography (CT) fracture patterns in these 24 patients (22 operative cases) were correlated with outcome (at least 2 years post-op with Musculoskeletal Functional Assessment (MFA) and Short-Form 36 (SF-36) as outcome measures). The study correlated fractures lines extending into the superior acetabulum with poorer prognosis, whereas fractures of the central acetabulum and quadrilateral surface were associated with a better prognosis. The same authors have demonstrated that the superior acetabular region and the posterior wall have the highest bone density in the acetabulum (Fig. 3) [16]. Today, three-dimensional CT scanning greatly facilitates the surgeon’s understanding: 1) insight into the importance of the fracture relative to the critical zones of the acetabulum (the roof and the posterior wall, as discussed above), 2) visualizing fracture planes to facilitate reduction and establish orthogonal screw fixation.

There are numerous studies demonstrating outcomes after non-operative treatment of acetabular fractures. In one such study, 57 patients were treated non-operatively with 7.9-year average follow-up; satisfactory results were demonstrated in 75% [17]. Poorest results were demonstrated in fractures involving the roof. If the displacement at the roof was < 2 mm, good to excellent (G-E) results were achieved.

Although nonsurgical treatment is a potential option in acetabular fractures, the past 30–40 years have seen a significant increase in open reduction internal fixation of these injuries. With a higher number of orthopaedic traumatologists in North America as well as improved trauma protocols, the majority of these cases are being performed at trauma centers with trained pelvic/acetabular surgeons.

The rest of this chapter will address factors which affect clinical outcome of patients who undergo operative treatment of displaced acetabular fractures. Two groups of factors affecting outcome will be discussed – pre-existing conditions and conditions at the time of injury.

## Preexisting conditions

Patient age, osteoporosis, and associated medical comorbidities affect the clinical outcome of operatively treated acetabular fractures.

### Age

With the aging population, the incidence of elderly patients with acetabular fractures has also increased markedly [18]. In a retrospective analysis of 53 patients with operatively treated acetabular fractures followed for 2 years, the authors found that age, fracture complexity, and damage to the femoral head were statistically significant predictors of negative outcome [19]. Patients younger than 40 years of age had a better prognosis than their older counterparts. The authors attribute poorer outcomes in older patients in part to osteoporosis, which presents challenges to surgical reduction and fixation.

In a larger study by Matta of 262 fractures followed for a minimum of 2 years, age was shown to be an independent risk factor for clinical outcome [20]. In patients younger than 40 years of age, 81% demonstrated a G-E results compared to only 68% of patients 40 years or older.

A more recent study sought to identify factors in patients 55 years or older that influenced radiographic and clinical outcomes [21]. In their cohort of 93 patients with a mean age of 67 years, the authors found fracture reduction, development of avascular necrosis, and previous contralateral total hip arthroplasty were statistically significant predictors for secondary surgeries. At an average of 5 years follow up the rate of conversion to total hip replacement was 30.95%.

**Fig. 4:**
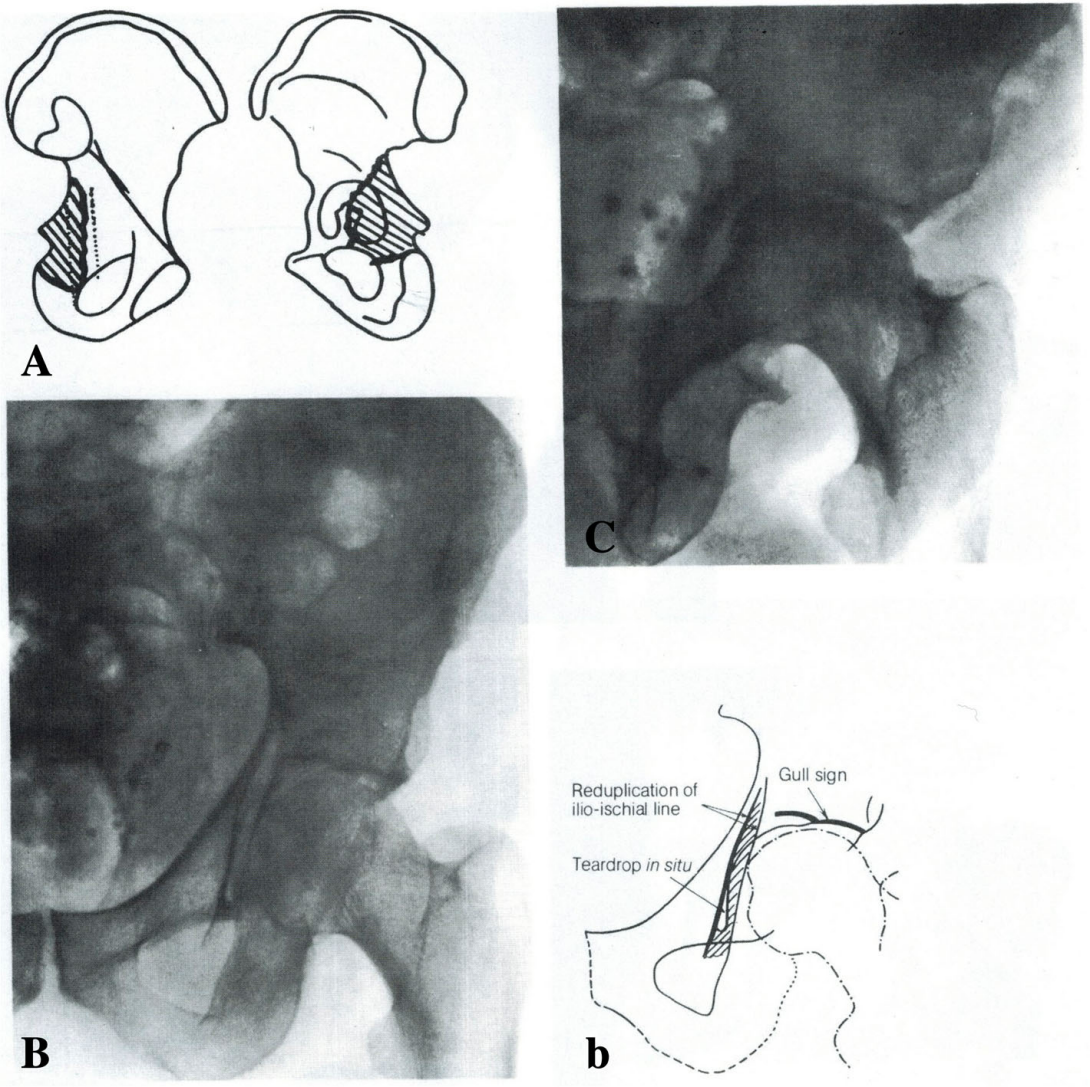
Line drawing and (**A**, b) and anteroposterior (**B**), iliac oblique (**C**) radiographs of the “gull-sign.” The fragment is a partial fracture of the posterior column (**A**) not including the ischial tuberosity; it involves the posterior portion of the quadrilateral surface, the ischial spine, and the roof is hinged inwards. This displacement of the posterior quadrilateral surface manifests as a re-duplication of the ilioischial line and the rotated roof segment, adjacent to the intact roof, appears as a gull in flight. Permission was granted for utilization of this figure in this review

The “gull sign” was initially described by Letournel and R. Judet and referred to a double roof arc outline in a posterior column fracture (Fig. 4, Letournel and R. Judet 1993) [4]. This particular fracture involves the angle of the greater sciatic notch and descends across the posterior portion of the quadrilateral surface including the ischial spine; the ischial tuberosity is not involved. This posterior column fragment is hinged inwards, and on an AP radiograph, the displaced hind portion of the quadrilateral surface is visualized as a reduplication of the ilioischial line. The portion of the roof that accompanies this posterior column fragment is also hinged inwards and gives an appearance, along with the intact roof, of a gull in flight. The “gull sign” has also been described in more recent literature [22], although in this article it was due to impaction of the *anteromedial* roof into osteopenic supporting bone, rather than the posterior column. In Anglen’s article, this anteromedial impaction of osteopenic bone was a poor prognostic sign.

Letournel described one patient with a complex anterior column fracture approached through the ilioinguinal incision; the “double-arc” sign on radiography was due to a fracture of the posterior portion of the acetabulum. The osteopenia did not allow a perfect reduction and the patient needed a total hip at one year post-operatively. These radiographic findings are important to mention since comminuted, osteoporotic fractures are more prevalent in our more aging population. There fractures that occur in acetabular zones prone to joint failure may be better treated with alternative methods such as limited open reduction internal fixation/total hip arthroplasty (ORIF/THA) or conservative treatment.

Others have sought to identify negative prognostic indicators for outcome in elderly cohorts. Zha et al., in a retrospective study of 86 patients, determined that femoral head injury and comminuted posterior wall fractures were negative prognostic indicators in the elderly [23]. Comminuted, osteoporotic fractures were not an independent predictor of poor outcome but rather an independent predictor of the quality of reduction. In these cases, consideration may be given to either the need to support these reductions with structural graft or whether the risk of surgery in these individuals outweighs the benefits (i.e. limited ORIF with a total hip replacement at a later date). While age alone is associated with worse radiographic and clinical outcomes, these additional factors may help to identify patients who are more likely to require a secondary surgery following operatively treated acetabular fracture. Further research may further delineate fracture patterns in the elderly which may be amenable to other treatment options than ORIF.

### Obesity

Obesity is a growing problem in the United States with predictions that over 50% of the population will be obese by 2030 [24]. With the negative impacts of obesity on health it follows that higher Body Mass Index (BMI) adversely affects postoperative complications and outcomes of operatively treated acetabular fractures. In a retrospective analysis of 169 consecutive operatively treated acetabular fractures patients were stratified based on BMI to assess postoperative complications [25]. The authors demonstrated that patients with a BMI > 30 were 2.1 times more likely to have an estimated blood loss of > 750 cc and 2.6 times more likely to have a deep vein thrombosis, while patients with a BMI > 40 were 5 times more likely to have a wound infection. Operative treatment of obese patients is sometimes unavoidable; however, we can use this information to inform patients of their increased risks of complications and investigate methods to mitigate them.

## Time of injury

Injury-related factors that impact clinical outcomes of acetabular fractures include fracture pattern, fractures with associated hip dislocation, and cartilage damage to the acetabulum and/or the femoral head.

### Fracture pattern

The classification of acetabular fractures by Letournel identified five elementary patterns in which a part or all of one column of the acetabulum is fractured and five associated patterns which include at least two of the elementary fractures [2]. By virtue of this, the associated patterns are viewed as more complex. In a large study of operatively treated acetabular fractures, Matta noted an anatomic reduction for 96% of elementary fracture patterns and 64% of associated fracture patterns [20]. All of the poor reductions in this study were of associated fracture patterns with T-shaped-posterior wall fractures having the highest prevalence of poor results. This finding was echoed in a report of 161 operatively treated acetabular fractures followed for 10 years [26]. The authors found certain fracture patterns were associated with a poor outcome and identified the T-shaped fracture with an associated posterior wall fracture as the “worst case scenario” as this fracture is both difficult to reduce and has a high rate of articular cartilage damage.

Despite this finding, though, many of the studies referenced in this chapter have differing outcomes for different fracture patterns. For example, although it is generally felt that associated fracture patterns may potentially be more difficult to reduce than elementary fracture patterns, this belief is not always the case. In the two largest outcome studies of operatively treated acetabular fractures [3, 4], anterior wall fractures had the worst prognosis. Comminuted posterior wall fractures, as will be discussed in the next section, also have a poor prognosis. The anterior wall, posterior wall, and superior zone of the acetabulum have the highest bone density due to the mechanical load vector. Anterior wall injuries tend to occur in individuals with osteoporotic bone; these fractures are difficult to reduce and prone to loss of reduction. Poor prognosis appears to correlate with joint destruction of these critical zones rather than discrete fracture patterns.

Outcomes for specific fracture patterns will be discussed. However, it should be noted that there are many confounding variables that may affect reported outcomes. Differences in 1) geographic location 2) surgeon experience, 3) fracture classifications, 4) choice of surgical approaches, 5) reduction and fixation techniques, 6) patient cohorts, and 7) clinical/radiographic assessment tools/analysis are all significant confounding variables in patient outcomes. Given these variables, though, we attempted to look at overall *trends*, or consensus findings, of these published outcomes.

#### Both-column acetabular fractures

Lichte et al., in a retrospective study of 115 both-column acetabular fractures demonstrated anatomical reduction the most important parameter for a good clinical outcome in these injuries [27]. Initial displacement of greater than 10 mm and presence of intra-articular fragments correlated with a negative clinical outcome. If two of the following three factors were present (femoral head dislocation, femoral head injury, or damage to acetabular joint surface), the patient was at greater risk for joint degeneration than if less than two of these factors were present. Although, in the presence of an anatomical reduction, the presence of two or more factors did not correlate with a negative outcome – this confirms that anatomic reduction is the most important factor in a successful outcome.

Gänsslen demonstrated 70% G-E clinical results in operatively treated both-column fractures with femoral head injury and/or initial articular surface displacement being negative prognostic indicators [28]. In any discussion of both-column acetabular fractures, the concept of secondary surgical congruence warrants discussion. Even in surgical fixation of both-column fractures, the surgeon may find that he/she cannot achieve anatomic reduction of the fracture. In both-column fractures, the free acetabular joint fragments may follow the femoral head superiorly and medially and may remain congruous around the femoral head in a displaced position relative to the pelvis – secondary surgical congruence [4]. In these cases, roof-arc measurements may reveal involvement of prognostically important portions of the acetabulum; however, these acetabular fracture lines may not be accurate since potential rotational displacement of the columns may lead to *perceived* gaps in the acetabulum with minimal loss of congruence. In these cases, if the fracture displacement is > 10 mm, as best assessed on CT, surgical intervention is warranted. An understanding of this concept of secondary surgical congruence is important in management of both-column fractures since it can affect treatment. Sometimes, reduction of the non-articular segment of the both-column fracture may be difficult. If the surgeon notes that there is secondary surgical congruence, though, he/she can minimize devoting time to factors which have marginal effect on outcome.

Numerous surgeons have demonstrated G-E results of both-column fractures if secondary surgical congruence is achieved [3, 4, 29]. The roof of the acetabulum is the most critical portion of the joint. The majority of the surgeon’s efforts should be dedicated to weighing out surgical or nonsurgical treatment options to maximize an excellent reduction of this portion of the joint with the least morbidity.

#### Posterior wall acetabular fractures

Depending on the cohort, posterior wall acetabular fractures may be one of the most common acetabular fracture patterns treated. Herman C. Epstein published some of the first outcome studies on this fracture pattern [30]. He demonstrated superior outcomes with removal of incarcerated fragments and open reduction internal fixation compared to closed reduction [31]. As discussed earlier, the roof and posterior wall of the acetabulum have the highest bone density due to the highest mechanical loads. Thus, injuries to these areas are prone to joint failure and anatomic reduction is critical.

Multiple authors have reported on their outcomes of surgical treatment of posterior wall acetabular fractures. Letournel reported 75% excellent results on 87 fractures [4]. Matta reported a 76% 20-year survivorship on 107 operatively-treated posterior wall acetabular fractures [3]. Pantazopolous et al. reviewed 52 posterior wall fractures 2–15 years after injury and also correlated fracture reduction with clinical/radiographic results with G-E clinical results in 85% [32]. Chiu et al. demonstrated 81% G-E results with an average follow-up of 7 years [33]. Mitsonis et al. published their results of associated posterior hip dislocations with posterior wall fractures with a mean follow-up of 18.5 years [34]. As suspected, they confirmed that clinical outcome correlated with fracture reduction (< 2 mm G-E results). They also found no correlation between time to reduction of the hip dislocation and incidence of avascular necrosis.

Despite the perceived “simplicity” of this fracture pattern, there is great potential for significant joint morbidity. Saterbak et al. demonstrated poor outcome associated with posterior wall comminution and fracture involvement of the subchondral arc [35]. In their study, all failed cases presented within one-year post-surgery with findings such as posterior head subluxation and narrowing of the superior joint space. In another review of 94 patients with operatively treated posterior wall fractures followed for 5 years the authors report 10.6% poor clinical outcome [36]. Reduction delay > 12 h after hip dislocation, age > 55, and extensive intra-articular comminution were factors associated with a poor clinical result.

Kreder et al. assessed the functional, clinical, and radiographic outcomes of 128 patients with simple and complex posterior wall fractures to identify factors associated with adverse outcome [37]. Severe functional deficits were determined by the MFA and SF-36 scores and correlated with the development of arthritis. Radiologic evidence of arthritis was present in 38.3% of patients at an average of 5.3 years follow up. Factors that correlated with arthritis included: 1) *radiographic evidence of arthritis*, 2) *associated fracture pattern of posterior wall with posterior column, 3) marginal impaction, and 4) residual displacement of > 2 mm*.

Moed confirmed these results in a study of 46 patients with elementary posterior wall fractures [38]. Similar results were noted with total MFA scores well below the normative values, indicating residual functional deficits persist following operatively treated posterior wall acetabular fractures. Clinical outcome can be poor despite anatomic reduction in posterior wall fractures and associated fracture patterns involving the posterior wall.

Like Moed, Matta reported 22 posterior wall fractures with an anatomical reduction yet only 68% of the patients reported G-E results [20]. Matta suggested that plain radiographs may not demonstrate articular incongruities. Moed further demonstrated that CT reveals articular incongruities better than plain radiographs and correlates better with clinical outcome [39]. Intra-operative fluoroscopy and the post-operative radiographs have been the benchmark of reduction evaluation. However, newer intra-operative fluoroscopic machines (O-arm™, Medtronic) that allow for 2-dimensional and 3-dimensional reconstructions may be beneficial to visualize joint incongruities. Still, a thorough understanding of intra-operative fluoroscopy is one of the most valuable skills to the acetabular surgeon. It is important to understand that the radiodensity of the subchondral bone is *maximized* when the x-ray beam is *tangential* to the curve of the acetabulum. This concept is especially important when utilizing oblique views of the acetabulum to assess joint reduction.

A retrospective cohort study by Firoozabadi et al. demonstrated that that posterior wall fracture fixed with less than 1 mm of diastasis/step-off based on CT had no conversion to a THA [40]. For fractures fixed with 1–4 mm of diastasis/step-off, there was a 10% conversion, and for 4 mm or more of malreduction, the conversion rate was 54%.

#### Anterior column/anterior wall fractures

There is sparse literature on isolated anterior column and/or anterior wall fractures except those noted in large series. Reduction of the anterior column with an association anterior wall is important. Even slight displacement of the fracture line in the anterior column can cause imperfection in the anterior wall reduction with subsequent joint incongruity.

Letournel mentioned that the anterior wall acetabular fracture demonstrates the least satisfactory results amidst the simple fracture patterns – 67% G-E results [4]. Matta demonstrated a 34% 20-year survivorship of operatively-treated anterior wall fractures [3]. Letournel attributed these results to the fact that these patients are often elderly with osteopenic bones. These fractures are not only difficult to reduce, but even after reduction, they are prone to loss of reduction. And since they involve the roof, joint failure is more probable.

Others have also examined the outcomes of anterior column/anterior wall acetabular fractures. In a study of 30 anterior column +/− anterior wall cases treated via an ilioinguinal approach (76%) or percutaneous techniques (24%), Giannoudis demonstrated 76% G-E results [41]. These results seem more favorable compared to Matta and Letournel’s. In this study by Giannoudis, though, there were only 4 anterior wall fractures of 30 – the rest of which were isolated anterior column fractures – which have a much more favorable prognosis.

Hessmann demonstrated 73–85% G-E functional results in his cohort of surgically treated anterior column fractures, but worse results with anterior wall fractures secondary to their incidence in elderly patients with osteoporotic bone [42].

#### Anterior column posterior hemitransverse acetabular fractures

No specific references could be identified examining outcomes after operative fixation of these fracture types except in the context of larger studies. These fracture patterns have similar outcomes to both-column acetabular fractures. This fracture pattern is essentially the same except it is “hinged” in the posterior column. Letournel achieved 82.2 and 85.3% G-E results in both column and anterior column/posterior hemitransverse operatively treated acetabular fractures [4]. Matta demonstrated 91 and 88% 10-year survivorship of both-column and anterior column/posterior hemitransverse acetabular fractures [3].

#### Transverse acetabular fractures

There is recent literature regarding outcomes after surgical fixation after transverse acetabular fractures. The outcomes correlate with involvement of the tectum, or the roof of the acetabulum, since transtectal transverse acetabular fractures have a worse outcome. Li et al. reported on outcomes following surgical fixation of 37 patients with 75% G-E results [43]. Positive outcomes correlated with radiographic outcomes. Poor outcomes were correlated to comminuted fractures of the roof, posterior hip instability, and damage to the femoral head. Oh et al. also correlated comminution of the roof with poor results [44].

#### Transverse plus posterior wall acetabular fractures

Numerous articles have demonstrated the propensity of the acetabulum with transverse fracture plus posterior wall injury to have less favorable results. Matta demonstrated 20-year survivorship of 74%. Letournel demonstrated 74.2% G-E results. Gänslenn reported on the results of 104 surgically treated patients with transverse plus posterior wall acetabular fractures [45]. He demonstrated 59.2% G-E results and joint failure in 32.7%. Joint failure was more likely in fractures with acetabular comminution. These findings agree with the aforementioned results by Oh et al. in which comminution of the dome portends a poor outcome.

#### T-shaped acetabular fractures

We could not identify any specific outcome studies on surgically treated T-shaped acetabular fractures. The authors can speculate, though, based on outcomes of other similar fracture patterns, i.e. transverse fractures, that clinical outcomes may correlate with involvement of the roof (trans-tectal types). Since, in transverse acetabular fractures, the hemipelvis hinges on the pubic symphysis, rotation is less of an issue since the surgeon can afford a direct reduction with the “assistance” of a stable base – the pubic symphysis. In T-shaped acetabular fractures, the ischio-pubic ramus segment is free-floating and in addition to restoring the roof, rotation of this segment can be difficult to reduce. Since the rotation of this segment will influence the joint, attention should be paid to the anatomic reduction.

#### Posterior column +/− posterior wall acetabular fractures

We could not identify any articles dedicated to clinical outcomes after surgical fixation of posterior column +/− posterior wall acetabular fractures. Nevertheless, we will discuss their clinical outcomes from large published series. Letournel demonstrated 81.82% excellent results in his cohort of 492 patients with posterior column fractures [4]. This rate drops to 29.4% when there is an associated posterior wall fracture. Matta demonstrated a 100% 20-year survivorship in a cohort of 14 operatively treated posterior column acetabular fractures [3]. When the posterior column fracture is associated with a posterior wall fracture, the 20-year survivorship decreases to 85% (26/816 fractures). Thus, despite the disparity in *outcomes*, the same trend is demonstrated in two of the largest-series: posterior column fractures have a better prognosis than posterior column plus posterior wall fractures. This concept is not surprising given the previous discussion on posterior wall fractures and their significant potential for joint failure.

### Posterior hip dislocation

Posterior hip dislocation is a controversial factor that has been implicated to have a negative prognosis in treatment of acetabular fractures. While some advocate for reduction within 24 h [46], Letournel did not feel time to reduction is an important determinant of outcome [4]; the rate of avascular necrosis in patients who had their hip reduced within six hours, 7–24 h, or 2–3 days was 5, 8, and 4%, respectively. The overall rate was 7.5%. They felt that the primary insult to the medial femoral circumflex arteries occurred at the time of injury. The greatest stretching of the medial femoral circumflex artery (MFCA) may occur in pure dislocation of the hip *without* fracture of the posterior wall. When a posterior wall fracture-dislocation occurs, the femoral head can potentially rest in the fracture defect – with the MFCA under less tension than resting “outside” the joint.

Bhandari’s results coincide with those of Letournel – demonstrating no significant association between time to relocation and radiological grade, clinical grade, or the development of arthritis [47]. Pantazopolous et al. also did not feel that time to reduction determined outcome; they also felt that the vascular insult occurs at the time of the accident and not during dislocation [32].

Others have demonstrated that time to posterior femoral head dislocation reduction does effect outcome. In a study of 94 patients with posterior wall acetabular fractures followed for a mean of 5 years, Moed identified delay in reduction of hip dislocation of greater than 12 h and age greater than 55 to be important prognostic factors for outcome [46]. In this same study, osteonecrosis of the femoral head and intra-articular comminution were also important determinants of outcome. The authors comment, though, that osteonecrosis does not always occur with delay in reduction of hip dislocation > 12 h, and furthermore, early reduction does not necessarily prevent this complication.

To further examine factors predictive of outcome following acetabular fracture with posterior hip dislocation, Bhandari and Matta reviewed 109 patients managed operatively within 3 weeks of injury with follow up of two or more years [47]. Dislocations were reduced at a median of 18 h from injury and all fractures were treated operatively. Anatomic reductions were achieved in 88% and G-E clinical outcomes in 84% of patients at an average of 5.9 years follow up (range 2–19). While quality of reduction, time to reduction of dislocation, and damage to the femoral head were all statistically significantly associated with radiologic grade, *the quality of the articular reduction* was the most important variable predictive of clinical outcome at follow up. Hip dislocations should be reduced as early as possible, but the ability to achieve an anatomic reduction should be the highest priority in surgical planning of these cases.

### Cartilage damage to the femoral head and/or acetabulum

Cartilage damage to the femoral head is another injury related factor that impacts functional outcome. Liebergall et al. reviewed 53 operatively treated acetabular fractures and found patient age younger than 40, simple fracture pattern, and absence of damage to the femoral head were statistically significant positive predictors of a positive outcome [19]. Damage to the femoral head was assessed on preoperative radiographs, CT scans, and at the time of surgery. In their series 26.4% of patients with femoral head cartilage damage went on to failure due to post-traumatic arthritis.

In Matta’s series of 262 fractures damage to the femoral head also affected outcome as 80% of patients without cartilage damage had a G-E result, while only 60% of patients with cartilage damage demonstrated a G-E result [20]. Similar findings were reported by Mears, with worse outcomes associated with impaction or abrasion of the femoral head or acetabulum [48]. A good-excellent clinical outcome was noted in 89% of patients with < 10% impaction. For those with 11–20% involvement, 70% progressed to a fair or poor outcome; all patients with 21–40% involvement demonstrated a poor outcome.

More recently, J. Clarke-Jenssen et al. demonstrated that injury to the femoral head and acetabular impaction as the strongest predictors of failure after acetabular fracture fixation [49]. When both of these factors were present, survival of the native hip fell to 0% at 3 years post-operatively in patients > 60 years of age.

Rommens et al. correlated presence of subchondral impaction, fracture comminution, and intra-articular fracture fragments with a negative outcome despite anatomical reduction [50]. These poor outcomes may explain cases with poor outcomes despite anatomic reduction. Cartilage damage to the femoral head/acetabulum and marginal impaction are predictors of poor clinical outcome.

## Surgical treatment

Table 1 is a summary of outcome studies of operative treatment of displaced acetabular fractures. This table demonstrates that, despite differences in surgeon, geographic location, fracture classification, surgical exposure/approach, reduction/fixation techniques, and radiographic/clinical outcome analysis, there are certain consensus prognostic indicators for outcomes. The vast majority of these outcomes studies in Table 1 possess one common theme that has been clearly demonstrated by Judet, Letournel, and Matta: *the accuracy of the fracture reduction is the strongest correlate with clinical outcome.* Even Pennal, in his series from 1957 to 1980 - despite the utilization of different surgical approaches - demonstrated a negative correlation between poor reduction, injury to the acetabular roof, age > 40, and clinical outcome [51]. Rather than discuss these studies in detail, we have focused on the two largest studies by Letournel and Matta [3, 4].

**Table 1:** Outcome studies of operative treatment of displaced acetabular fractures listed in order of number of cases surgically treated with follow-up, year of publication, country of origin, number of cases, average follow-up period (F/U yrs), G-E results OR survivorship (% survival at 10 or 20 yrs), and negative prognostic factors

Author	Year	Country	Cases (F/U yrs)	G-E Result/Survivorship	Negative prognostic factors
Tannast/Matta et al [3]	2012	USA	816 (2–20)	85% (10YR) 79% (20 YRS)	FHI, PW, AGE, DISP, MI
Letournel/Judet [4]	1993	FRANCE	492 (1–33)	80%	PC/PW, AW, PR
Mears et al [48]	2003	USA	424 (9.3)	89%	PR, FN, DEL > 11, AF, SI, FHI, OB, AW, AGE
Matta [20]	1996	USA	255 (6)	76%	AGE, FHI, SI, TT/PW
Clarke-Jenssen et al [49]	2017	NORWAY	253 (12)	86% (10YRS)	FHI, SI
Madhu et al [53]	2006	UK	237 (2.9)	76%	DEL > 15 (EF), DEL > 10(AF)
Murphy et al^a^ [65]	2003	IRELAND	180 (6.3)	78%	AF, AGE, PR > 3, HO, LC
Rommens et al [66]	1997	BELGIUM	175 (2)	76%	TT/PW
Mayo [67]	1994	USA	163 (3.7)	75%	–
Briffa et al [26]	2011	UK	161 (11.3)	72%	AGE, DEL > 15, PR, PC/TT, FHI
Pennal et al^a^ [51]	1980	CANADA	103 (7.25)	–	FX, WB, PR, AGE, PELVIS
Wright et al [56]	1994	USA	87 (3.6)	45%	DL, HO, AVN, AGE, PR, EXP
Zha et al^b^ [23]	2013	CHINA	86 (3.2)	84%	CPWF, FHI, PR
Fica et al [68]	1998	CHILE	84 (5.5)	67%	TT, PR, AGE, AVN
Zhi et al [69]	2011	CHINA	82 (2.8)	71%	FX, AGE, LE FX, PR, DEL, DL
Rommens et al [50]	2011	GERMANY	77 (3.7)	70%	CPWF, SI, IAF
Almedia et al [70]	2011	BRAZIL	76 (4.9)	81%	PR, LOR, DI
Deo et al [71]	2001	UK	74 (2.6)	74%	FH, PR, NERVE/DL
Chen et al [72]	2000	TAIWAN	73 (7.5)	74%	PR
Uchida et al [73]	2013	JAPAN	71 (8.6)	90%	PR, AVN, SI
Ragnarsson et al [74]	1992	SWEDEN	55 (15)	60%	PR
Heeg et al [75]	1990	HOLLAND	54 (9.6)	61%	PR, HO
Kebaish et al [54]	1991	CANADA	54 (4.7)	86%	EXP, PR
Ruesch/Mast et al [76]	1994	USA	53 (1+)	81%	N/A
De Ridder et al [55]	1994	HOLLAND	51 (3)	76%	–
Oranksy et al [77]	1993	ITALY	50 (3.5)	76%	DEL > 21, PR, EXP
Chiu et al^c^ [33]	1996	CHINA	27 (7)	81%	–
Brueton [78]	1993	UK	26 (2.2)	61%	PR, DEL > 17

*G-E results* good to excellent results, *FHI* femoral head injury, *CPWF* comminuted posterior wall fragment, *AW* anterior wall fracture, *PR* poor reduction, *SI* subchondral impaction, *IAF* intra-articular fragment, *FX* fracture pattern, *WB* damage to wb dome, *PELVIS* injury to the pelvic ring, *AGE* patient age > 40, *FN* ipsilateral fem. neck fx, *AF* associated fx, *EF* elementary fx, *EXP* surgeon experience, *DEL* delay to surgery (i.e. delay > 15 days), *TT* t-shaped acetabular fracture, *PW* posterior wall acetabular fracture, *DI* deep infection, *LOR* loss of reduction, *NERVE* nerve injury, *DL* dislocation, *LE FX* lower extremity fracture, *OB* obesity, *HO* heterotopic ossification, *LC* local complications

^a^ This study utilized different surgical approaches

^b^ Cohort of elderly patients

^c^ Cohort of operatively treated posterior wall fractures

Letournel demonstrated 80.69% good-very good-excellent results in 492 hips treated surgically within 3 weeks of injury as assessed by the method of D’Aubigne and Postel [52].

- Despite 94% perfect reductions of posterior wall fractures, 79.5% achieved at least a very good result. He felt this discrepancy was due to 1) osteonecrosis and 2) posterior wall comminution.
- Among the associated fracture patterns, the worst outcome group was the posterior column/posterior wall group (29.41% excellent results) – followed by transverse/posterior wall (48.51% excellent results).
- Among the simple fracture patterns, the least satisfactory results occurred among anterior wall fractures (67%) – which he accounted for because these fractures often occurred in elderly individuals with osteopenic bone.
- Out of 302 cases with perfect radiographic results, 283 (93.2%) had perfect intra-operative reductions and 293 had excellent clinical results; a very good clinical result corresponds to a perfect radiographic appearance in 98.6% of cases
- Imperfect reductions treated conservative/operative treatment may still lead to good/very good functional results if the femoral head remains congruent to a segment of the articular surface large enough to withstand the increased intra-articular pressure. Patients with surgical secondary congruence achieved 56% very good and 24% good results.
- Complications in his series were: mortality 2.3%, post-operative infection 4.2%, post-operative sciatic palsy 6.3%, avascular necrosis 4.5%, post-traumatic arthritis 19.7%, ectopic bone formation 28.2%

Matta demonstrated 79% 20-year survivorship in 816 patients following open reduction internal fixation of displaced acetabular fractures (2–20 year follow-up) [3]. Table 2 demonstrates survivorship according to fracture type and other characteristics.

**Fig. 5:**
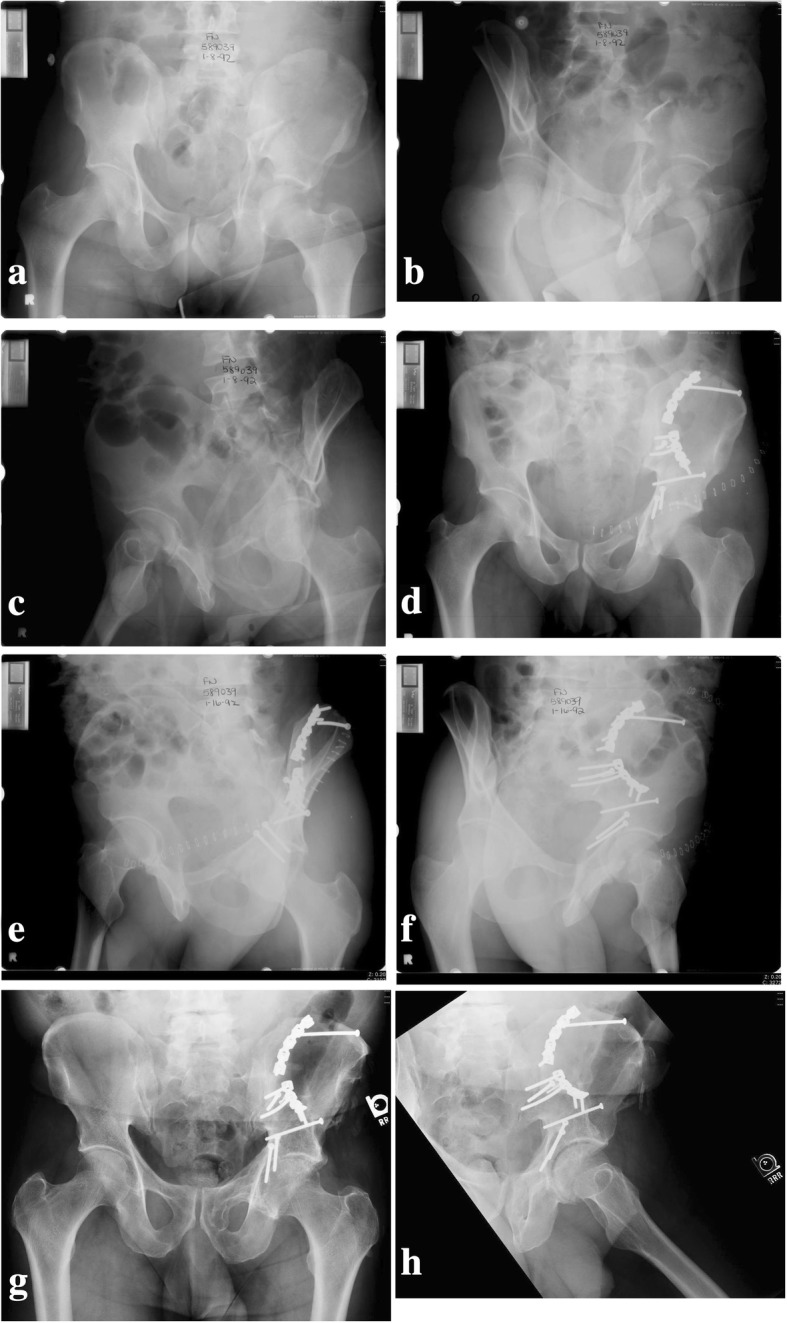
Pre-operative (**a**–**c**), immediate post-operative (**d**), and 21-year post-operative radiographs (**e**–**h**) of a patient after reduction and fixation of a both-column acetabular fracture. At 21 years, the left hip superior joint space is intact and patient has a good clinical outcome

- Of those 816 surgeries, the reduction was categorized as 0–1 mm in 616 (75%), 2–3 mm in 148 (18%), and > 3 mm in 36 (4%).
- In this study, 20-year survivorship was poorest for anterior wall fractures (34%) and highest for transverse (89%) and both-column (87%) acetabular fractures.
- Overall rate of anatomical reduction increased from 40% in 1980 to 92% in 2006.
- Non-controllable independent predictors of negative outcome include: 1) Age > 40, 2) anterior dislocation, 3) femoral head cartilage lesion, 4) involvement of the posterior wall, 5) marginal impaction, and 6) initial displacement > 20 mm.
- Controllable independent predictors of negative outcome include: 1) nonanatomic reduction, 2) post-operative incongruence of the acetabular roof, and 3) utilization of the extended iliofemoral approach.
- A higher rate of anatomic reduction was noted in elementary fracture patterns, patients treated early (< 21 days) and patients younger than 40.
- Similar to other studies mentioned in this chapter (56) the first 50% of hip that failed did so by 1.5 years post-operatively. From 1.5 years to 20 years post-operatively, there is a linear decrease in survivorship
- Anterior wall acetabular fractures had a higher prevalence in in the elderly, were associated with marginal impaction, and more difficult to reduce.
- Both-column fractures had a significantly better outcome at twenty years, despite nonanatomic reduction – possibly due to secondary surgical congruence as the innominate bone potentially serves as a “crumple zone” and absorbs much of the energy rather than the cartilage of the femoral head. Figure 5 demonstrates pre-operative, immediate post-operative, and 21-year post-operative radiographs of a patient after reduction and fixation of a both-column acetabular fracture.

**Table 2:** Survivorship of operatively-treated acetabular fractures according to fracture type and other characteristics

	Survivorship (95% Confidence Interval) (%)				
	Two Years	Five Years	Ten Years	Twenty Years	Median Time to Failure
Entire series (*n* = 816)	91 (90–92)	88 (87–90)	85 (84–87)	79 (76–81)	1.5
Elementary fracture type (*n* = 241)	91 (89–93)	86 (84–89)	84 (81–87)	73 (68–79)	1.3
Anterior wall (*n* = 12)	91 (82–100)	68 (53–84)	68 (53–84)	34 (9–59)	2.3
Anterior column (*n* = 80)	95 (92–97)	92 (88–95)	87 (83–91)	77 (70–85)	3.0
Posterior wall (*n* = 107)	88 (84–91)	82 (78–86)	81 (77–85)	76 (71–82)	1.2
Posterior column (*n* = 14)	100	100	100	100	–
Transverse (*n* = 28)	89 (83–95)	89 (83–95)	89 (83–95)	89 (83–95)	0.3
Associated fracture type (*n* = 575)	92 (91–93)	89 (88–91)	86 (84–87)	80 (78–83)	1.6
Posterior column, posterior wall (*n* = 26)	85 (78–92)	85 (78–92)	85 (78–92)	85 (78–92)	0.5
Transverse, posterior wall (*n* = 143)	89 (86–91)	85 (82–88)	81 (78–85)	74 (68–80)	1.5
T-shaped (*n* = 96)	89 (85–92)	85 (81–89)	77 (72–81)	74 (65–84)	1.6
Ant. column, post. Hemitrans. (*n* = 76)	92 (89–95)	92 (89–95)	88 (84–92)	75 (65–84)	1.3
Both columns (*n* = 234)	96 (94–97)	83 (91–95)	91 (89–93)	87 (83–90)	2.2
Initial displacement					
≥ 20 mm (*n* = 226)	86 (84–89)	84 (81–86)	78 (75–81)	68 (63–73)	1.3
≤ 20 mm (*n* = 590)	93 (92–95)	90 (89–91)	88 (86–89)	83 (81–85)	1.9
Treatment delay					
< 21 days (*n* = 730)	93 (92–94)	89 (88–91)	86 (85–88)	79 (77–82)	2.0
> 21 days (*n* = 86)	82 (78–86)	80 (75–84)	74 (69–79)	74 (69–79)	0.9
Previous surgery					
Yes (*n* = 5)	60 (38–82)	30 (6–54)	–	–	0.8
No (*n* = 811)	92 (91–93)	89 (87–90)	85 (84–87)	79 (77–81)	1.6
Age					
< 40 yr. (*n* = 386)	96 (95–97)	95 (94–96)	92 (91–94)	87 (84–89)	2.3
40–65 yr. (*n* = 318)	88 (86–90)	83 (81–86)	81 (79–83)	74 (71–77)	1.3
> 65 yr. (*n* = 112)	83 (79–87)	79 (75–83)	70 (65–76)	51 (38–64)	0.8
> 75 (*n* = 42)	80 (73–87)	74 (66–83)	65 (54–76)	–	0.6

Per Letournel:

*“One single factor appears paramount: the relocation of the head under a sector of roof of sufficient extent must be adequate. This is the practical prerequisite for all good results. However, it must not be taken that obtaining this result obviates the need for good reduction of the columns supporting the acetabulum.”*

Even with an anatomic reduction on post-operative radiographs, small displacements may exist. The surgeon should strive for an anatomic reduction since even a “perfect” reduction to his/her eyes will likely have incongruities which may affect clinical outcome. If anatomic reduction and secure fixation is achieved, there is a higher probability that the patient will not require further surgery. Matta’s study has demonstrated the value of ORIF up to 20 years+; at 20–30 years post-op, hip arthritis may still develop but may occur even if there was no injury.

## Time of surgery

Timing of surgery has also been identified as a factor that affects outcome with delay to surgery resulting in worse radiographic and functional outcomes. In a retrospective review of 237 patients Madhu et al. analyzed time to surgery as a variable and its effect on ability to obtain an anatomic reduction and functional outcome [53]. The odds of obtaining an anatomic reduction and G-E functional outcome decrease significantly as time to surgery increases. For patients with elementary fracture patterns a delay of 15 days was statistically significant for ability to achieve an anatomic reduction. For patients with associated fracture patterns a delay of only 5 days reached statistical significance.

In Mears’ study of 424 displaced acetabular fractures (both elementary and associated fractures) in 411 patients the authors sought to correlate outcome with multiple factors including time to surgery [48]. All patients were treated within 21 days and had a minimum of 3 years follow up. For patients treated operatively in the first 2 days the rate of anatomic reduction was 76%. For patients treated at 3–10 days the rate of anatomic reduction was 68%, and for patients treated at 11–21 days only 54% achieved anatomic reduction. Beyond 11 days there was a statistically significant decrease in the rate of anatomic reduction.

Similar results were reported by Matta in 262 fractures treated within 21 days [20]. The number of anatomic reductions decreased as time to surgery increased. For patients treated operatively in the first 7 days, 74% had an anatomic reduction. The rate of anatomic reduction decreased only slightly to 71% for patients treated between 8 and 14 days, however a significant decline was noted after 15–21 days with only 57% achieving anatomic reductions.

Based on the available data, the ability to achieve an anatomic reduction in a displaced acetabular fracture decreases significantly beyond 2 weeks. Since reduction correlates with functional outcome it is advisable to proceed with surgery early, within the first 14 days.

## Surgeon experience

Matta and Merritt [11], Kebaish [54], and de Ridder [55] have all demonstrated that surgeon experience also has a direct correlation with patient outcome in surgical treatment of these injuries. Letournel attributed his improved outcomes later in his career to surgeon experience. Others have noted worse results when less-experienced/trained surgeons treat acetabular fractures. Wright et al. demonstrated 45% G-E clinical outcomes after surgical treatment of 87 displaced acetabular fractures; they attributed these less-than-favorable results to surgeries being performed by 13 different orthopaedic surgeons [56]. This group felt surgeon experience was an important component to treating these fractures.

## Soft tissue

The potential role of the surrounding musculature and soft tissue, while not a direct cause of hip arthritis, can affect functional outcome after acetabular fractures. Hip muscle weakness after operative fixation of acetabular fractures has been shown to have an increased incidence in those patients with post-traumatic arthritis – independent of surgical approach [57]. Patients treated via either anterior or posterior approach for an acetabular fracture have significant alterations in their gait, muscle strength, and functional outcome [58]. Based on these findings, there may be a role for hip muscles in joint remodeling and/or functional outcome. Fernandez et al. have shown that muscle forces influence pelvis stress distribution and remodeling however, this study did not include analysis of the piriformis, obturator internus, or obturator externus – the “rotator cuff” of the hip [59]. Although not an evidence-based negative prognostic indicator, hip muscle/soft tissue compromise may affect functional outcome after acetabular fractures.

## Complications

Complications of acetabular fracture surgery include infection, nerve injury, heterotopic ossification, thromboembolic issues, malunion, and nonunion. These are discussed in depth in preceding chapters, however brief mention here is warranted. Rather than summarize complications of all the previously mentioned outcome studies, we will refer to the meta-analysis performed by Giannoudis et al. in 2005 [60].

### Infection

Based on available data, the incidence of infection is approximately 2–5%. Giannoudis meta-analysis determined a 4.4% local wound infection incidence in 2547 patients [60]. The risk of infection is increased with the presence of a soft-tissue degloving injury, the Morel-Lavallé lesion, with positive cultures in over 40% of cases [61].

### Iatrogenic nerve injury

Sciatic nerve palsy occurs more commonly with the posterior surgical approach with the peroneal division at greatest risk. Rates of iatrogenic sciatic nerve injury vary in the literature. Some surgeons have reported a 2% incidence of sciatic nerve injury with intraoperative visualization and protection of the nerve [62]. Letournel, as mentioned above, had a 6.3% incidence of post-operative sciatic nerve palsy [4]. Prior to distal femur traction with the knee flexed, his incidence was 18.4%. He attributed the decrease to less tension on the nerve (hip extension/knee flexion, careful placement of retractors with attention paid to retractor effect on nerve tension). Out of 34 cases with motor impairment, 9 completely recovered and 21 had significant recovery.

Giannoudis’ meta-analysis of 2426 fractures had an incidence of approximately 4.7% iatrogenic sciatic nerve palsy [60].

### Heterotopic ossification

The rate of heterotopic ossification is reported in up to 80% of cases treated with the posterior surgical approach [63]. While the use of radiation has reduced the rate of heterotopic ossification following posterior or extensile approaches, in certain cases the formation of heterotopic bone may become clinically significant requiring additional surgery to regain hip range of motion. In Giannoudis’ meta-analysis of 2394 displaced fractures, HO incidence was 25.6% with Brooker grade III or IV in 5.7% [60]. In this same study, the incidence of HO was 24.4% in the prophylaxis group (indomethacin, local radiation, or both) versus 25.7% in the non-prophylaxis group.

### Thromboembolic complications

Giannoudis demonstrated an incidence of 4.3% deep vein thrombosis (DVT) or pulmonary embolus (PE) out of 806 patients [60]. In these studies, there was inconsistent documentation of prophylaxis against DVT.

### Avascular necrosis

In a meta-analysis done of 2010 patients done by Giannoudis, AVN incidence was 5.6% [60]. Patients who sustain a posterior dislocation have a statistically significant higher incidence of AVN (9.2%) than those who did not (5%). Wear, either of the femoral head and/or acetabulum, is a sequelae of AVN. Wear can cause shortening of the extremity, limp, and spine/knee pain. Radiographic differentiation of AVN and post-traumatic arthritis can be difficult. Advanced stages of AVN may radiographically present with fragmentation and collapse.

AVN is also a potentially confounding variable when analyzing results. For example, the surgeon may achieve an anatomic reduction but a secondary surgery (i.e. total hip arthroplasty) may still be indicated due to AVN. Despite excellent clinical and radiographic reduction, the clinical outcome after AVN can still be poor. This point is especially important in posterior wall acetabular fractures with dislocation.

### Post-traumatic arthritis

Giannoudis meta-analysis result of severe grade III or IV OA was 19.1% - similar to Letournel’s incidence of 19.7% [60]. If the reduction was < 2 mm, the incidence was 13.2% but increases to 43.5% if the reduction was > 2 mm. Like AVN, wear of the femoral head and/or acetabulum is also a complication of post-traumatic arthritis, but discerning the difference between the two diagnoses can be difficult.

## Future directions

There have been numerous advances in pelvic and acetabular surgery including:

1. the advance of implant and radiographic technology
2. the significant increase in the dissemination of knowledge via the Internet and international courses
3. the vast increase in the number of trained orthopaedic trauma surgeons in the United States.

Despite these advances, however, we, as surgeons, are still struggling to improve our outcomes for surgical treatment of acetabular fractures. In this particular specialty of surgery, no technology can substitute for the human brain - the surgeon’s 3-dimensional understanding of the biological approach, the bony anatomy, the fracture pattern, and the reduction and fixation techniques via the exposure. And despite the increase in education, there is no substitute for experience in treating these injuries. Unfortunately, with the increase in the number of orthopaedic trauma surgeons at Level 1 and Level 2 trauma centers, there is no longer a large volume of experience for any one surgeon.

Nevertheless, those surgeons who have significant experience in treating these injuries may ponder whether we have reached our maximum potential to achieve a “macroscopic” anatomic reduction? In other words, even in the best of hands, have we reached a 70–80% G-E outcome ceiling given current techniques, reduction tools, technology, and knowledge of acetabular fractures? Others have also questioned whether well-trained acetabular surgeons have reached their limits and now limited by the cartilage biology, genetics, and/or other unknown factors [26].

**Fig. 6:**
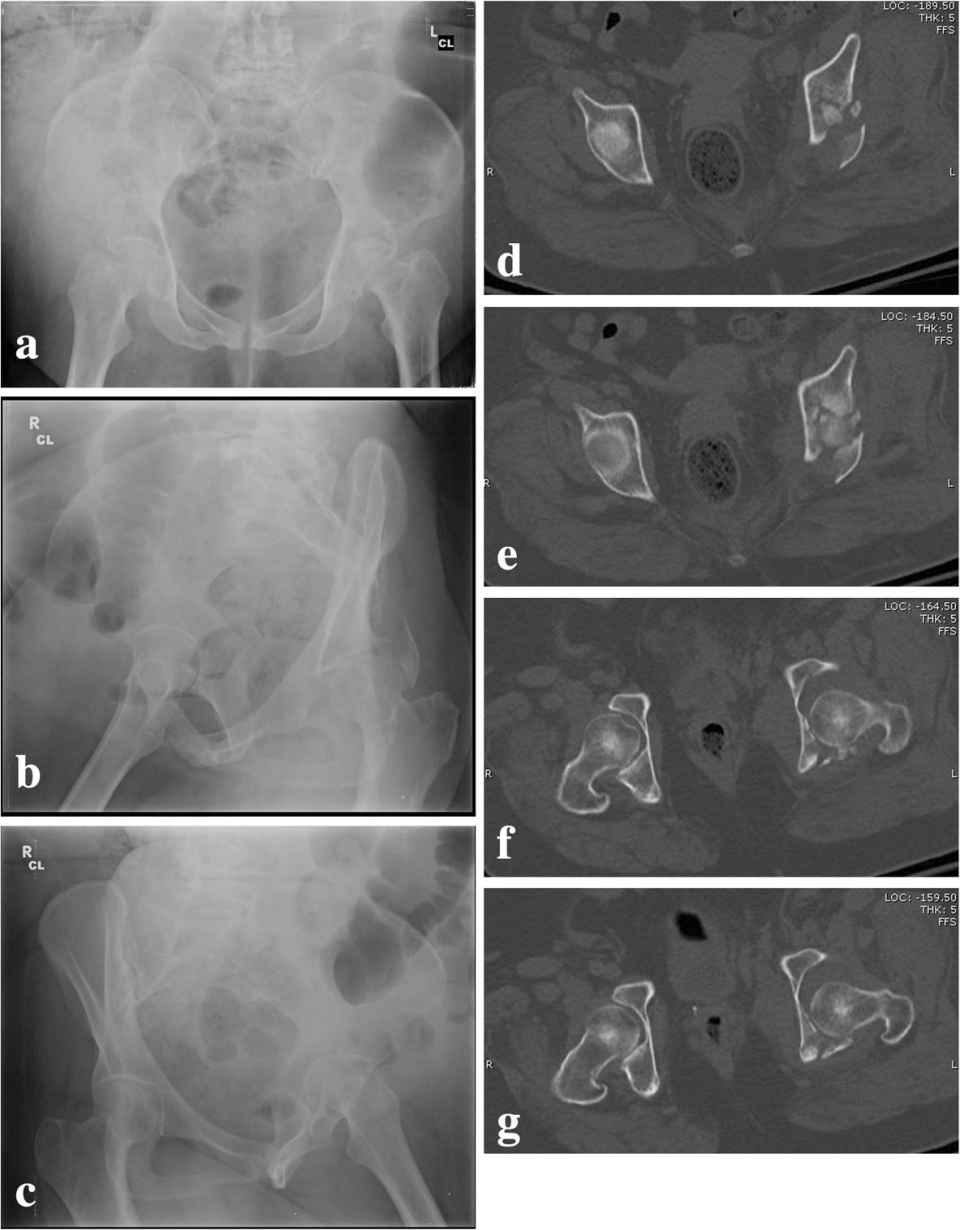
Pre-operative (**a**–**c**) radiographs and CT (**d**–**g**) of a patient who sustained a comminuted transtectal transverse acetabular fracture with involvement of the posterior wall

**Fig. 7:**
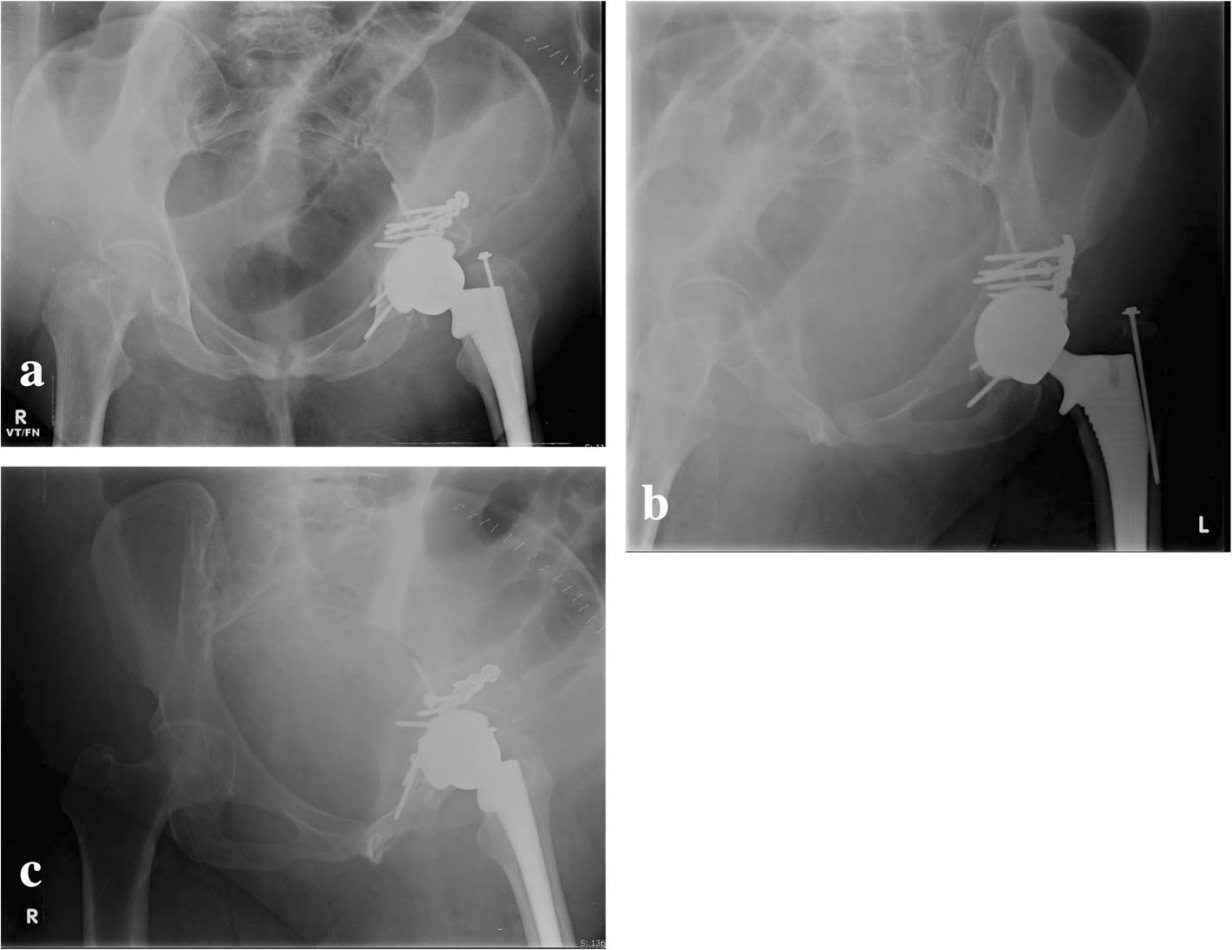
Post-operative AP pelvis (**a**) and Judet view (**b**–**c**) radiographs of a transtectal + posterior wall acetabular fracture after limited open reduction internal fixation and acute total hip arthroplasty

For the rest of us though, the future will still remain in understanding the fundamental principles of acetabular surgery. The principles introduced by Judet, Letournel, and Matta have yielded positive clinical outcomes and have stood the test of time. Current and future emphasis should be on understanding these fundamental exposures and reduction techniques since they have proven their value to pelvic/acetabular surgeons around the world. More importantly, these fundamental principles must not be forgotten or the future will be spent re-learning the past. Newer innovations such as reduction techniques, instrumentation, and operating tables should build upon, rather than replace, the fundamental principles. Past surgeons from one hundred years ago likely never envisioned a future in which the injured acetabulum could be restored to near normal with operative intervention through anatomic “windows.” Future efforts in our understanding of acetabular fractures may focus on the following:

1. *Obtaining and maintain reduction*
  Past statistics indicate this priority. Thus, future efforts should be on achieving and maintaining reduction such as improving ease of implant placement and improved reduction tools. However, there is great potential in assistance from operating room tables and motor/robotic devices.
2. *Pre- and intra-operative radiographic understanding of the injured acetabulum*
  A deeper and more quantitative pre-operative knowledge of the prognostic areas of the injured acetabulum based on radiographs and further 3-dimensional CT scan analysis; this understanding may be patient specific due to significant genetic variation in femoral head shape, femoral neck/acetabular anteversion, acetabular coverage, and other individual-specific biomechanical factors.
  Intra-operative mobile 2D/3D imaging systems, such as the O-arm**™** by Medtronic, may be a beneficial technology to more accurately assess joint fracture reduction and implant position.
3. *Indications*
  As mentioned previously, future studies may delineate specific fracture patterns in select individuals that are more appropriately treated with ORIF vs. THA vs. conservative treatment. For example, comminuted transtectal transverse fractures, especially with involvement of the posterior wall (Fig. 6), may be better treated with limited ORIF and primary THA (Fig. 7).
4. *Bone substitutes/cartilage biology*
  Bone graft substitutes may help achieve and maintain reduction and fixation – especially in osteoporotic individuals. In time, surgical intervention may be augmented by therapies based on current molecular biological research. Over the past 3–4 decades, this research has improved greatly improved our understanding of cartilage and tidemark regeneration. Lastly, inflammatory factors that may predispose to joint failure are being identified [64]; this orthogenomic insight may ultimately be translated into new therapeutics.

Surgical treatment of acetabular fractures may not be for everyone. There are many intricacies of acetabular surgery that are still poorly understood by many novice and experienced surgeons alike: fracture classification, how to properly read the X-rays, an understanding of the fracture planes, performing a proper exposure, achieving an anatomic reduction, understanding screw trajectories in a bone with complex 3-dimensional orientation, and intra-operative and post-operative reduction assessment. Many of these concepts take years of experience to understand; and, in an era where there are more pelvic/acetabular surgeons, there simply may not be enough volume to go around.

Ideally, acetabular surgery should not be regarded as “getting ready for a total hip replacement” or “preparation for future reconstructive surgery.” Letournel, Judet, and Matta have developed a methodology to restore native hip biomechanics with long survivorship when performed properly. Thus, apart from select fracture patterns in select cohorts, all efforts should be made to strive for anatomic reduction and restore native biomechanics. Some fracture patterns’ complexity may be too difficult for some surgeons. In the best interest of the patient, these complex cases should be transferred to a more experienced and skilled acetabular surgeon.

As mentioned before, there is significant variation in methodologies, reduction techniques, operative approaches, and definitions of “excellent” reduction/outcome. Efforts should be made to stick to proven principles and outcome assessment tools previously set forth. This consistency allows for more uniform comparison of results and potentially more accurate statistics.

Despite current technological progress, we should still strive for improvement and further insight - especially in the management of injuries complex as acetabular fractures. Letournel’s critical insight into his own mistakes and dedication to improvement are reflected in his own writings. His efforts were focused on a deep understanding of fundamental truths and the communication of these concepts to others - rather than self-promotion based on false truths.

This “confident humility” is an important essence for any surgeon to embrace.

## Conclusions

Surgical treatment of acetabular fractures is challenging. Since initial studies by Letournel and Judet, numerous groups have published their results on clinical outcomes after treatment of these injuries. Based on the data presented in this chapter, the following factors are negative prognostic indicators for clinical outcome after surgical fixation of acetabular fractures:

1. Patient age > 40
2. Poor fracture reduction (> 3 mm)
3. Multi-fragmentary fractures of the posterior/anterior wall
4. Transverse multi-fragmentary fractures of the tectum
5. Cartilage damage to the femoral head and/or acetabulum
6. Delay to surgery > 5 days and > 15 days for associated and elementary fractures patterns, respectively.
7. Initial fracture displacement > 20 mm

Despite these consensus findings, surgical principles established by Judet, Letournel, and Matta can yield 80% good-excellent outcome results. However, 50 % of joints that fail will do so within the first two years after surgical fixation.

Anatomic reduction is the most influential factor predictive of clinical outcome and is what surgeons should strive for in the treatment of these fractures. With the increase in orthopaedic pelvic/acetabular surgeons and the strong relation between anatomic reduction and clinical outcome, emphasis should be on sound exposure and reduction techniques for these injuries.

## Abbreviations

AVN: Avascular necrosis
BMI: Body mass index
CT: Computed tomography
DVT: Deep vein thrombosis
G-E: Good to excellent
MFA: Musculoskeletal Functional Assessment
ORIF: Open reduction internal fixation
PE: Pulmonary embolus
SF-36: Short-Form 36
THA: Total hip arthroplasty
